# 1-Benzoyl-*N*-phenyl­cyclo­propane­carboxamide

**DOI:** 10.1107/S1600536808034077

**Published:** 2008-10-25

**Authors:** Wen-Liang Li, Zhi-Guo Zhu

**Affiliations:** a Experimental Center of Medicine, Jilin Medical College, Jilin 132013, People’s Republic of China

## Abstract

The title compound, C_17_H_15_NO_2_, was synthesized by reaction of 1,2-dibromo­ethane with 1-benzoyl-*N*-phenyl­cyclo­propane­carboxamide and K_2_CO_3_ in dimethyl­formamide. The mol­ecule exhibits a V-shaped conformation in the crystal with a dihedral angle of 88.7 (3)° between the two benzene rings. Pairs of N—H⋯O hydrogen bonds link the mol­ecules into dimers about centres of inversion.

## Related literature

For further synthesis details, see: Zhang *et al.* (2007 ▶).
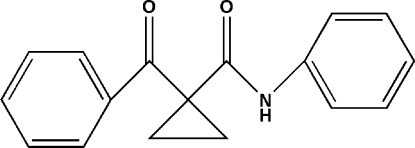


## Experimental

### 

#### Crystal data

C_17_H_15_NO_2_
*M* *_r_* = 265.30
Triclinic, 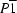

*a* = 7.424 (1) Å
*b* = 9.473 (1) Å
*c* = 10.831 (2) Å
α = 94.276 (2)°
β = 99.313 (2)°
γ = 105.773 (2)°
*V* = 717.72 (17) Å^3^
*Z* = 2
Mo *K*α radiation
μ = 0.08 mm^−1^
*T* = 293 (2) K
0.32 × 0.24 × 0.21 mm

#### Data collection

Bruker APEX CCD diffractometer
Absorption correction: multi-scan (*SADABS*; Sheldrick, 1996 ▶) *T*_min_ = 0.981, *T*_max_ = 0.986
4053 measured reflections
2748 independent reflections
2213 reflections with *I* > 2σ(*I*)
*R*_int_ = 0.012

#### Refinement

*R*[*F* ^2^ > 2σ(*F* ^2^)] = 0.044
*wR*(*F* ^2^) = 0.119
*S* = 1.04
2748 reflections
185 parameters
1 restraint
H atoms treated by a mixture of independent and constrained refinement
Δρ_max_ = 0.15 e Å^−3^
Δρ_min_ = −0.15 e Å^−3^

### 

Data collection: *SMART* (Bruker, 1997 ▶); cell refinement: *SAINT* (Bruker, 1997 ▶); data reduction: *SAINT*; program(s) used to solve structure: *SHELXS97* (Sheldrick, 2008 ▶); program(s) used to refine structure: *SHELXL97* (Sheldrick, 2008 ▶); molecular graphics: *SHELXTL* (Sheldrick, 2008 ▶); software used to prepare material for publication: *SHELXTL*.

## Supplementary Material

Crystal structure: contains datablocks global, I. DOI: 10.1107/S1600536808034077/bi2312sup1.cif

Structure factors: contains datablocks I. DOI: 10.1107/S1600536808034077/bi2312Isup2.hkl

Additional supplementary materials:  crystallographic information; 3D view; checkCIF report

## Figures and Tables

**Table 1:** Hydrogen-bond geometry (Å, °)

*D*—H⋯*A*	*D*—H	H⋯*A*	*D*⋯*A*	*D*—H⋯*A*
N1—H1*N*⋯O1^i^	0.879 (14)	2.03 (1)	2.896 (1)	167.0 (1)

Symmetry code: (i) 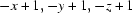.

**Fig. 1.**
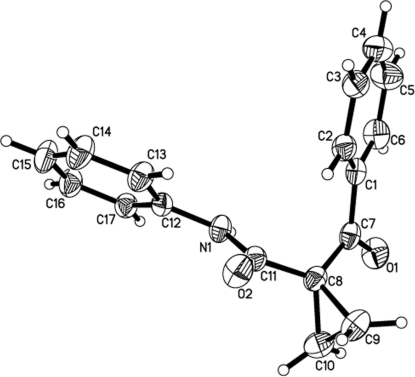
Molecular structure of the title compound with displacement ellipsoids drawn at the 30% probability level for non-H atoms.

## supplementary crystallographic information

### Comment

Derivatives of 1-benzoyl-*N*-phenylcyclopropanecarboxamide have been long known for their diverse pharmacological and biological properties. The method for the synthesis of similar compounds has been reported previously (Zhang *et al.*, 2007). In the crystal, the title compound exhibits a "V-shaped" conformation with interplanar angle of 91.3 (3)° between the two benzene rings (C1–C6 and C12–C17). The C8–C10 cyclopropane ring makes dihedral angles of 120.8 (3)° and 125.9 (2)° with the C1–C6 and C12–C17 benzene rings, respectively. The value of the dihedral angle between the planes defined by C7—O1—C8 and the C1–C6 ring is 18.8 (3)°.

### Experimental

1,2-Dibromoethane (0.95 ml, 11 mmol) was added to a solution of 1-benzoyl-*N*-phenylcyclopropanecarboxamide (2393 mg, 10 mmol) and K_2_CO_3_ (2950 mg, 23 mmol) in DMF (25 ml) and the mixture was stirred for 10 h (monitored by TLC) before being slowly poured into ice-water (200 ml). The precipitated white solid was filtered and purified by flash silica gel column chromatography (eluent: ether/ethyl acetate (1/3)) to give the title compound as colourless crystals.

### Refinement

H atoms bound to C atoms were placed geometrically and refined using a riding model with C—H = 0.93 Å for aromatic H or 0.97 Å for CH_2_ groups, and with *U*_iso_ = 1.2*U*_eq_(C). The H atom of the NH group was located in a difference Fourier map and refined with the N—H distance restrained to be 0.88 (1) Å and with *U*_iso_(H) = 1.5*U*_eq_(N).

### Crystal data

**Table:**

C_17_H_15_NO_2_	*Z* = 2
*M**_r_* = 265.30	*F*(000) = 280
Triclinic, *P*1	*D*_x_ = 1.228 Mg m^−^^3^
Hall symbol: -P 1	Mo *K*α radiation, λ = 0.71069 Å
*a* = 7.424 (1) Å	Cell parameters from 2748 reflections
*b* = 9.4730 (13) Å	θ = 1.9–26.0°
*c* = 10.8310 (15) Å	µ = 0.08 mm^−^^1^
α = 94.276 (2)°	*T* = 293 K
β = 99.313 (2)°	Block, colourless
γ = 105.773 (2)°	0.32 × 0.24 × 0.21 mm
*V* = 717.72 (17) Å^3^	

### Data collection

**Table:**

Bruker APEX CCD diffractometer	2748 independent reflections
Radiation source: fine-focus sealed tube	2213 reflections with *I* > 2σ(*I*)
graphite	*R*_int_ = 0.012
ω scans	θ_max_ = 26.0°, θ_min_ = 1.9°
Absorption correction: multi-scan (SADABS; Sheldrick, 1996)	*h* = −6→9
*T*_min_ = 0.981, *T*_max_ = 0.986	*k* = −11→11
4053 measured reflections	*l* = −13→13

### Refinement

**Table:**

Refinement on *F*^2^	Secondary atom site location: difference Fourier map
Least-squares matrix: full	Hydrogen site location: inferred from neighbouring sites
*R*[*F*^2^ > 2σ(*F*^2^)] = 0.044	H atoms treated by a mixture of independent and constrained refinement
*wR*(*F*^2^) = 0.119	*w* = 1/[σ^2^(*F*_o_^2^) + (0.0561*P*)^2^ + 0.1075*P*] where *P* = (*F*_o_^2^ + 2*F*_c_^2^)/3
*S* = 1.04	(Δ/σ)_max_ < 0.001
2748 reflections	Δρ_max_ = 0.15 e Å^−^^3^
185 parameters	Δρ_min_ = −0.15 e Å^−^^3^
1 restraint	Extinction correction: SHELXL97 (Sheldrick, 2008), Fc^*^=kFc[1+0.001xFc^2^λ^3^/sin(2θ)]^-1/4^
Primary atom site location: structure-invariant direct methods	Extinction coefficient: 0.054 (6)

### Special details

**Table:**

Geometry. All e.s.d.'s (except the e.s.d. in the dihedral angle between two l.s. planes) are estimated using the full covariance matrix. The cell e.s.d.'s are taken into account individually in the estimation of e.s.d.'s in distances, angles and torsion angles; correlations between e.s.d.'s in cell parameters are only used when they are defined by crystal symmetry. An approximate (isotropic) treatment of cell e.s.d.'s is used for estimating e.s.d.'s involving l.s. planes.
Refinement. Refinement of *F*^2^ against ALL reflections. The weighted *R*-factor *wR* and goodness of fit *S* are based on *F*^2^, conventional *R*-factors *R* are based on *F*, with *F* set to zero for negative *F*^2^. The threshold expression of *F*^2^ > σ(*F*^2^) is used only for calculating *R*-factors(gt) *etc*. and is not relevant to the choice of reflections for refinement. *R*-factors based on *F*^2^ are statistically about twice as large as those based on *F*, and *R*- factors based on ALL data will be even larger.

### Fractional atomic coordinates and isotropic or equivalent isotropic displacement parameters (Å^2^)

**Table:**

	*x*	*y*	*z*	*U*_iso_*/*U*_eq_	
C1	0.4980 (2)	0.72180 (15)	0.70564 (13)	0.0477 (4)	
C2	0.4788 (2)	0.72747 (17)	0.83128 (14)	0.0511 (4)	
H2	0.5289	0.6680	0.8832	0.061*	
C3	0.3862 (2)	0.82028 (18)	0.87980 (16)	0.0603 (4)	
H3	0.3762	0.8246	0.9644	0.072*	
C4	0.3088 (3)	0.9062 (2)	0.8034 (2)	0.0735 (5)	
H4	0.2463	0.9689	0.8361	0.088*	
C5	0.3235 (3)	0.8996 (2)	0.6782 (2)	0.0811 (6)	
H5	0.2691	0.9569	0.6263	0.097*	
C6	0.4180 (3)	0.8089 (2)	0.62925 (16)	0.0658 (5)	
H6	0.4283	0.8059	0.5447	0.079*	
C7	0.6119 (2)	0.63374 (16)	0.65224 (13)	0.0498 (4)	
C8	0.6665 (2)	0.51699 (16)	0.72240 (13)	0.0488 (4)	
C9	0.8739 (2)	0.5593 (2)	0.78679 (19)	0.0733 (5)	
H9A	0.9546	0.6558	0.7773	0.088*	
H9B	0.9038	0.5258	0.8680	0.088*	
C10	0.8063 (3)	0.4471 (2)	0.67465 (19)	0.0736 (5)	
H10A	0.7951	0.3450	0.6874	0.088*	
H10B	0.8459	0.4749	0.5967	0.088*	
C11	0.5257 (2)	0.41812 (16)	0.78762 (13)	0.0471 (4)	
C12	0.1879 (2)	0.26815 (15)	0.74941 (13)	0.0455 (3)	
C13	0.1551 (2)	0.27890 (19)	0.87138 (15)	0.0591 (4)	
H13	0.2398	0.3500	0.9333	0.071*	
C14	−0.0041 (3)	0.1833 (2)	0.90016 (17)	0.0742 (5)	
H14	−0.0252	0.1893	0.9824	0.089*	
C15	−0.1321 (3)	0.0793 (2)	0.80958 (19)	0.0748 (5)	
H15	−0.2386	0.0148	0.8303	0.090*	
C16	−0.1014 (3)	0.07135 (19)	0.68809 (17)	0.0661 (5)	
H16	−0.1887	0.0021	0.6260	0.079*	
C17	0.0577 (2)	0.16516 (16)	0.65724 (15)	0.0525 (4)	
H17	0.0775	0.1592	0.5746	0.063*	
O1	0.67064 (19)	0.66389 (14)	0.55579 (10)	0.0730 (4)	
O2	0.56848 (17)	0.38898 (14)	0.89348 (11)	0.0682 (4)	
N1	0.34958 (18)	0.36333 (14)	0.71454 (11)	0.0503 (3)	
H1N	0.338 (3)	0.368 (2)	0.6331 (14)	0.076*	

### Atomic displacement parameters (Å^2^)

**Table:**

	*U*^11^	*U*^22^	*U*^33^	*U*^12^	*U*^13^	*U*^23^
C1	0.0472 (8)	0.0443 (8)	0.0446 (8)	0.0012 (6)	0.0084 (6)	0.0072 (6)
C2	0.0540 (9)	0.0509 (8)	0.0491 (8)	0.0119 (7)	0.0149 (7)	0.0123 (7)
C3	0.0634 (10)	0.0598 (10)	0.0608 (10)	0.0154 (8)	0.0239 (8)	0.0097 (8)
C4	0.0724 (12)	0.0700 (12)	0.0874 (14)	0.0306 (10)	0.0227 (10)	0.0127 (10)
C5	0.0869 (14)	0.0849 (14)	0.0832 (14)	0.0441 (12)	0.0096 (11)	0.0250 (11)
C6	0.0737 (12)	0.0677 (11)	0.0518 (9)	0.0153 (9)	0.0059 (8)	0.0150 (8)
C7	0.0493 (8)	0.0519 (8)	0.0394 (7)	−0.0013 (7)	0.0113 (6)	0.0034 (6)
C8	0.0442 (8)	0.0519 (8)	0.0472 (8)	0.0079 (6)	0.0127 (6)	0.0010 (6)
C9	0.0484 (10)	0.0856 (13)	0.0772 (12)	0.0084 (9)	0.0073 (9)	0.0070 (10)
C10	0.0663 (12)	0.0811 (13)	0.0827 (13)	0.0263 (10)	0.0321 (10)	0.0088 (10)
C11	0.0494 (9)	0.0471 (8)	0.0447 (8)	0.0116 (7)	0.0118 (6)	0.0066 (6)
C12	0.0470 (8)	0.0430 (7)	0.0479 (8)	0.0111 (6)	0.0141 (6)	0.0101 (6)
C13	0.0560 (10)	0.0667 (10)	0.0486 (9)	0.0058 (8)	0.0153 (7)	0.0028 (7)
C14	0.0724 (12)	0.0884 (13)	0.0582 (10)	0.0057 (10)	0.0307 (9)	0.0116 (9)
C15	0.0653 (11)	0.0684 (12)	0.0818 (13)	−0.0052 (9)	0.0304 (10)	0.0107 (10)
C16	0.0602 (10)	0.0549 (10)	0.0724 (11)	−0.0010 (8)	0.0165 (9)	−0.0017 (8)
C17	0.0556 (9)	0.0496 (8)	0.0514 (8)	0.0108 (7)	0.0156 (7)	0.0046 (7)
O1	0.0927 (9)	0.0769 (8)	0.0522 (7)	0.0144 (7)	0.0360 (6)	0.0146 (6)
O2	0.0616 (7)	0.0863 (9)	0.0541 (7)	0.0149 (6)	0.0070 (5)	0.0249 (6)
N1	0.0510 (7)	0.0538 (7)	0.0413 (7)	0.0039 (6)	0.0123 (6)	0.0091 (6)

### Geometric parameters (Å, °)

**Table:**

C1—C6	1.388 (2)	C9—H9B	0.970
C1—C2	1.390 (2)	C10—H10A	0.970
C1—C7	1.488 (2)	C10—H10B	0.970
C2—C3	1.378 (2)	C11—O2	1.2118 (17)
C2—H2	0.930	C11—N1	1.3566 (19)
C3—C4	1.370 (3)	C12—C13	1.383 (2)
C3—H3	0.930	C12—C17	1.383 (2)
C4—C5	1.376 (3)	C12—N1	1.4161 (18)
C4—H4	0.930	C13—C14	1.376 (2)
C5—C6	1.375 (3)	C13—H13	0.930
C5—H5	0.930	C14—C15	1.372 (3)
C6—H6	0.930	C14—H14	0.930
C7—O1	1.2196 (16)	C15—C16	1.371 (2)
C7—C8	1.495 (2)	C15—H15	0.930
C8—C11	1.5080 (19)	C16—C17	1.378 (2)
C8—C10	1.510 (2)	C16—H16	0.930
C8—C9	1.514 (2)	C17—H17	0.930
C9—C10	1.476 (3)	N1—H1N	0.879 (14)
C9—H9A	0.970		
			
C6—C1—C2	118.62 (15)	H9A—C9—H9B	114.8
C6—C1—C7	118.78 (14)	C9—C10—C8	60.95 (11)
C2—C1—C7	122.46 (13)	C9—C10—H10A	117.7
C3—C2—C1	120.68 (15)	C8—C10—H10A	117.7
C3—C2—H2	119.7	C9—C10—H10B	117.7
C1—C2—H2	119.7	C8—C10—H10B	117.7
C4—C3—C2	120.01 (16)	H10A—C10—H10B	114.8
C4—C3—H3	120.0	O2—C11—N1	124.09 (13)
C2—C3—H3	120.0	O2—C11—C8	122.85 (14)
C3—C4—C5	119.93 (18)	N1—C11—C8	113.04 (12)
C3—C4—H4	120.0	C13—C12—C17	119.77 (14)
C5—C4—H4	120.0	C13—C12—N1	121.74 (14)
C6—C5—C4	120.52 (17)	C17—C12—N1	118.46 (13)
C6—C5—H5	119.7	C14—C13—C12	119.32 (16)
C4—C5—H5	119.7	C14—C13—H13	120.3
C5—C6—C1	120.21 (17)	C12—C13—H13	120.3
C5—C6—H6	119.9	C15—C14—C13	121.14 (16)
C1—C6—H6	119.9	C15—C14—H14	119.4
O1—C7—C1	119.69 (14)	C13—C14—H14	119.4
O1—C7—C8	120.33 (14)	C14—C15—C16	119.35 (16)
C1—C7—C8	119.80 (12)	C14—C15—H15	120.3
C7—C8—C11	120.13 (13)	C16—C15—H15	120.3
C7—C8—C10	117.63 (13)	C15—C16—C17	120.55 (16)
C11—C8—C10	114.96 (13)	C15—C16—H16	119.7
C7—C8—C9	114.24 (13)	C17—C16—H16	119.7
C11—C8—C9	116.43 (14)	C16—C17—C12	119.85 (15)
C10—C8—C9	58.41 (12)	C16—C17—H17	120.1
C10—C9—C8	60.64 (11)	C12—C17—H17	120.1
C10—C9—H9A	117.7	C11—N1—C12	126.26 (12)
C8—C9—H9A	117.7	C11—N1—H1N	118.1 (12)
C10—C9—H9B	117.7	C12—N1—H1N	114.0 (12)
C8—C9—H9B	117.7		
			
C6—C1—C2—C3	−1.6 (2)	C7—C8—C10—C9	102.84 (16)
C7—C1—C2—C3	174.30 (14)	C11—C8—C10—C9	−106.87 (16)
C1—C2—C3—C4	1.2 (3)	C7—C8—C11—O2	−135.19 (16)
C2—C3—C4—C5	0.1 (3)	C10—C8—C11—O2	75.3 (2)
C3—C4—C5—C6	−1.0 (3)	C9—C8—C11—O2	9.8 (2)
C4—C5—C6—C1	0.6 (3)	C7—C8—C11—N1	46.30 (18)
C2—C1—C6—C5	0.6 (3)	C10—C8—C11—N1	−103.20 (16)
C7—C1—C6—C5	−175.38 (16)	C9—C8—C11—N1	−168.75 (14)
C6—C1—C7—O1	17.1 (2)	C17—C12—C13—C14	2.1 (3)
C2—C1—C7—O1	−158.80 (15)	N1—C12—C13—C14	−179.83 (16)
C6—C1—C7—C8	−167.79 (14)	C12—C13—C14—C15	−1.1 (3)
C2—C1—C7—C8	16.4 (2)	C13—C14—C15—C16	−0.5 (3)
O1—C7—C8—C11	−144.49 (15)	C14—C15—C16—C17	1.0 (3)
C1—C7—C8—C11	40.38 (19)	C15—C16—C17—C12	0.1 (3)
O1—C7—C8—C10	4.2 (2)	C13—C12—C17—C16	−1.7 (2)
C1—C7—C8—C10	−170.91 (14)	N1—C12—C17—C16	−179.76 (14)
O1—C7—C8—C9	69.84 (19)	O2—C11—N1—C12	1.7 (2)
C1—C7—C8—C9	−105.29 (16)	C8—C11—N1—C12	−179.78 (13)
C7—C8—C9—C10	−108.67 (15)	C13—C12—N1—C11	34.8 (2)
C11—C8—C9—C10	104.33 (16)	C17—C12—N1—C11	−147.15 (15)

### Hydrogen-bond geometry (Å, °)

**Table:**

*D*—H···*A*	*D*—H	H···*A*	*D*···*A*	*D*—H···*A*
N1—H1N···O1^i^	0.88 (1)	2.03 (1)	2.896 (1)	167 (1)

Symmetry codes: (i) −*x*+1, −*y*+1, −*z*+1.
